# Analysis of Influencing Parameters Enhancing the Plucking Efficiency of Piezoelectric Energy Harvesters

**DOI:** 10.3390/s23063069

**Published:** 2023-03-13

**Authors:** Saša Zelenika, Petar Gljušćić, Andrea Barukčić, Marko Perčić

**Affiliations:** 1 University of Rijeka, Faculty of Engineering, Vukovarska 58, 51000 Rijeka, Croatia; 2 University of Rijeka, Centre for Micro- and Nanosciences and Technologies, Radmile Matejčić 2, 51000 Rijeka, Croatia; 3 University of Rijeka, Centre for Artificial Intelligence and Cybersecurity, Radmile Matejčić 2, 51000 Rijeka, Croatia

**Keywords:** energy harvesting, frequency up-conversion, additive manufacturing, DoE, experimental assessment, autonomous sensor nodes

## Abstract

The integration of energy harvesting systems into sensing technologies can result in novel autonomous sensor nodes, characterized by significant simplification and mass reduction. The use of piezoelectric energy harvesters (PEHs), particularly in cantilever form, is considered as one of the most promising approaches aimed at collecting ubiquitous low-level kinetic energy. Due to the random nature of most excitation environments, the narrow PEH operating frequency bandwidth implies, however, the need to introduce frequency up-conversion mechanisms, able to convert random excitation into the oscillation of the cantilever at its eigenfrequency. A first systematic study is performed in this work to investigate the effects of 3D-printed plectrum designs on the specific power outputs obtainable from FUC excited PEHs. Therefore, novel rotating plectra configurations with different design parameters, determined by using a design-of-experiment methodology and manufactured via fused deposition modeling, are used in an innovative experimental setup to pluck a rectangular PEH at different velocities. The obtained voltage outputs are analyzed via advanced numerical methods. A comprehensive insight into the effects of plectrum properties on the responses of the PEHs is attained, representing a new and important step towards the development of efficient harvesters aimed at a wide range of applications, from wearable devices to structural health monitoring systems.

## 1. Introduction

**Figure 1:**
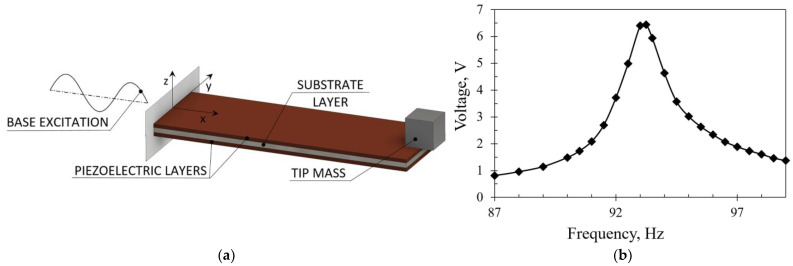
Bimorph PEH with a tip mass: graphical representation (**a**) and typical response when subjected to harmonic excitation (**b**) [2].

The process of energy harvesting (EH), i.e., collecting low-level ambient energy and converting it into usable electrical energy, is increasingly being studied as an efficient way to power autonomous sensing devices to be used, e.g., in wearable technologies, remote sensors, aircraft structural health monitoring (SHM) systems and numerous other settings [1,2,3,4]. The hence collected electrical energy can be used or stored using supercapacitors, and managed by employing an appropriate power management system [3,5,6]. The most promising energy sources available in the environment and suitable for EH transduction include solar or artificial light energy; waste heat, generated by most mechanical and biological systems; radio frequency (RF), caused by the emissions from communication networks; and kinetic energy, present in all moving systems, such as mechanical, biological as well as atmospheric [7,8]. For each of these energy forms, a specific technology is employed to collect and convert it into usable electricity, i.e., photovoltaics is used for collecting light energy, thermoelectric generators convert waste heat via the Seebeck effect, while specialized miniaturized antennas can be used to convert ambient electromagnetic radiation, such as RF waves [9,10,11,12,13]. In order to collect and convert the ubiquitous and copious kinetic energy, among several possible mechanisms, such as the electromagnetic or the electrostatic energy harvesters, one of the most commonly used EH transduction principles is the bimorph cantilever, comprising two layers of piezoelectric material, electromechanically coupled via the 31 mode, attached to a typically metallic substrate (Figure 1a) [5,6].

The piezoelectric energy harvester (PEH) is herein clamped at one end, while its free end can oscillate freely. A tip mass can be attached to the free end enabling the tuning of the device’s eigenfrequency to a specific ambient excitation and amplifying the resulting deformations, thus increasing the voltage output. The excitation can be induced via the clamped base (harmonic excitation) and by plucking or impacting the free end (plucking/impact excitation). As the conversion efficiency of a cantilever PEH is highest when the excitation frequency matches its coupled electromechanical eigenfrequency, the highest voltage (and power) outputs are generated at these exact operating conditions [5,6,14]. A typical voltage output of a cantilever PEH under harmonic excitation is shown in Figure 1b.

The described phenomenon is particularly relevant if a PEH device, subjected to base excitation, is to be utilized for collecting kinetic energy from human motion, due to the random nature of such excitation [14,15,16]. Studies have, in fact, shown that the kinetic energy from human motion during the activities of daily living (ADLs), particularly from arm movement and footfalls, can result in a maximal power output of ~60 W [17], but the frequencies of the thus occurring excitations typically randomly range from 1 to 4 Hz, with relatively low acceleration values of <1 g [14,15,16,18]. This results, in turn, in a less than optimal operation of the cantilever PEH, and therefore, in a rather limited power output [6,14].

In order to overcome this limitation, several approaches have been suggested in the literature [19,20,21,22,23], such as the flywheel-based plucking or impacting of the PEH free end [19], the use of magnetic excitation [20], multiple cantilevers or nonlinear geometries [21], active tuning of the PEHs [22] or nonlinear force customization technologies [24]. One of the most promising approaches is the frequency up-conversion (FUC) mechanisms, i.e., the conversion of random ambient motion into periodical plucking excitation of the PEH, that induces the deflection (bending) of the free end of the cantilever and suddenly releases it, allowing the cantilever to freely oscillate [14,15,19]. Such an excitation mechanism generally comprises a flywheel collecting the random kinetic energy from the environment, equipped with single or multiple plectra aimed at inducing the excitation of the free end of the PEH. The thus plucked device is left to freely oscillate at its eigenfrequency, enabling its optimal operation, i.e., the highest conversion efficiency, regardless of ambient excitation [25].

Based on the well-known theoretical foundations relative to the responses of oscillatory systems to nonperiodic (impulsive, step or ramp—as in the plucking case) excitations [26], the complex mechanism of plucking PEH excitation has been studied to some extent in the recent literature [27,28]. The most relevant works focused on the effects of plucking parameters on the PEH response, i.e., the thickness and overlap of a compliant plectrum [27], or the duration of the applied force using an infinitely large mass simulating a stiff plectrum [28], providing a deeper understanding of the plucking mechanism along with valuable insights for the development of FUC-based devices. An indication that the parameters of the plucking mechanism, such as the mechanical properties, the size, the stiffness or the materials used to produce the plectra, as well as the plucking speed, can have a significant influence on the response of a plucked PEH, was, therefore, clearly provided.

Due to their outstanding rapid prototyping and cost-effective characteristics, additive manufacturing (AM) technologies are increasingly employed in EH setups [29,30]. Even though AM has been utilized in energy harvester manufacturing, especially for depositing PZT and Polyvinylidene fluoride (PVDF) layers [5,8,31,32,33], a systematic investigation of the influence of 3D-printed components on FUC-based PEH excitation has not yet been carried out. In this work, the readily available and cost-effective fused deposition modeling (FDM) AM variant is, therefore, considered as a viable mean of producing the plectra that are a critical element in plucked kinetic harvesting systems. The specific issues innate to this technological process, i.e., layer delamination, geometric accuracy issues, or anisotropy due to parts orientation and filament material, then make an in-depth study of these effects on PEHs’ responses necessary.

To broaden the understanding of all the cited influencing design parameters on the efficiency of plucked piezoelectric energy harvesting devices, a thorough experimental study focused on FDM manufactured plectra is, therefore, carried out in this work. The considered plectrum design configurations are, thus, described in Section 2, whereas the used novel experimental setup, aimed at exciting the PEHs via the FUC mechanisms, is explained in Section 3. Initial results are outlined in Section 4, while a thorough analysis of the obtained output powers vs. the variable design parameters and excitation velocities is given in Section 5. The conclusions are finally drawn in Section 6, where an outlook for future work is also provided.

## 2. Plectrum Geometry and DoE

To better understand the impact of the abovementioned effects, a new systematically structured experimental study, based on the design-of-experiments (DoE) methodology, is conducted in this work, analyzing the geometry and material of plectra produced by using the FDM AM technology to be used to excite the PEHs via the FUC mechanism.

**Figure 2:**
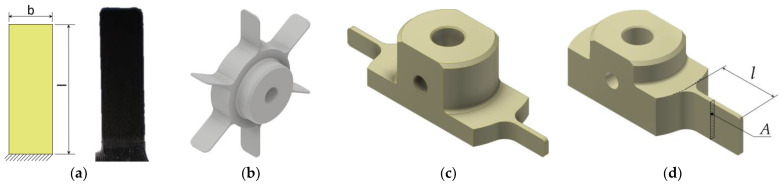
Considered rectangular plectrum shape (**a**), rotor equipped with six plectra (**b**) [34] and example of a two-plectra motor [15] (**c**), as well as the single-plectrum rotor design used in this work (**d**).

Different plectrum shapes were initially considered, but for some of them (triangular and elliptical) the actual 3D-printed geometry differed appreciably from the respective computer-aided design (CAD) models, strongly affecting their stiffness [26], while their tips were soon chipped away. The study converged, therefore, on the rectangular plectrum shape (Figure 2a), manufactured as part of a multi-plectrum rotor (Figure 2b), enabling the controlled rotation and the resulting periodical plucking PEH excitation [34].

On the other hand, due to the mentioned issues of AM, a notable variation of mechanical properties often occurs in 3D-printed objects with respect to their position and orientation during the manufacturing process [15,35]. This implies that a modification of the plectrum arrangement on the rotor must be adopted to achieve more consistent mechanical properties, and therefore, only two plectra (later reduced to a single one—see Section 5) are printed around the perimeter of the rotor (Figure 2c,d).

As the geometry of the plectrum, as well as the utilized material, strongly affect its stiffness, the variables considered in this study are then the plectrum cross-section, the length of the plectrum and the material used to produce it (see the symbols reported in Figure 2). The somewhat limited dimensional accuracy of the small-scale 3D-printed plectra limits the possibility of plectrum thickness variations; thorough investigation of this effect allowed establishing that the thickness can be consistently maintained at *t* = 700 ± 15 μm so that this thickness is adopted and kept constant for all the herein-studied plectra [34]. The plectrum width (less affected by the AM accuracy issues) is, in turn, altered to vary the resulting second moment of area of the cross-section. The inclusion of the plectrum rotation speed as an additional variable then enables an even more comprehensive investigation of the herein studied plucking FUC mechanism.

The performed study is, therefore, focused on the effects of the variation of several plectrum design parameters, i.e., the plectrum material, the second moment of area of the cross-section *I*_x_, as well as the plectrum length *l*, along with the rotating speed *n* of the plectrum carrying rotor (influencing the plucking conditions) on the plucked response of a conventional rectangular PEH. To facilitate the analysis of the obtained results, as well as to reduce the number of required experiments, a DoE methodology is used. The Latinized centroid Voronoi tessellation (LCVT) DoE variant [36,37,38] is, thus, applied to the considered set of variables, generating random combinations of their values. In the initial studies [34] (see Section 4), the parameters *l* and *I*_x_ defining the plectrum geometry, as well as the rotating speed *n* of the DC motor generating via the plectra the plucking excitation, are defined as continuous variables, while a selection of four different used polymeric plectrum materials is specified as a discrete variable. The main part of the study presented in this work focuses, in turn, on a single FDM material that showed the best performances in the initial studies, eliminating, thus, one of the DoE variables.

## 3. Experimental Setup and Plectrum Manufacturing

Based on the considerations outlined in Section 2, an experimental setup suited to perform FUC plucking PEH excitations with plectra of various designs and materials is presented in this section. The FDM AM technology used to produce the plectra themselves is also described.

### 3.1. Plucking Excitation Setup

**Figure 3:**
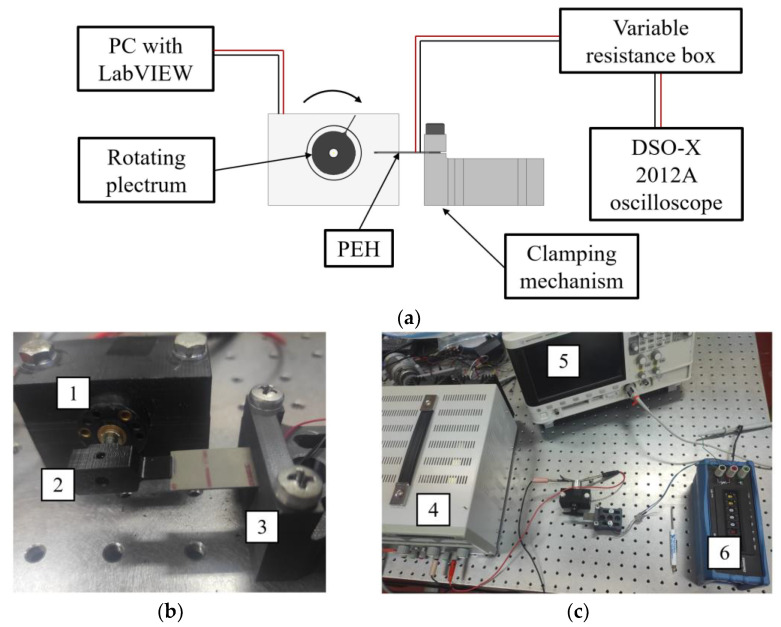
Experimental setup for plucking FUC PEH excitation: (**a**) schematic representation [14], (**b**) close-up of its actual execution showing the DC motor (1), the rotor with the plectrum (2) and the clamping mechanism with a PEH (3) and (**c**) the overall setup with the motor power supply (4), the oscilloscope (5) and the resistance box simulating the electrical load (6).

To enable the study outlined in Section 2, a suitable experimental setup, enabling the plucking excitation of a PEH, is devised, as schematically depicted in Figure 3a. The actual implementation of the setup, shown in detail in Figure 3b,c, comprises a Faulhaber motor-gearhead assembly of a 2233-S series DC motor paired with a 22E series gearhead with an *i* = 28:1 reduction ratio (1) [39]. The motor is equipped with an exchangeable rotor (2) enabling the study of different plectra exciting the PEHs via the FUC mechanism, while a rigid 3D-printed clamping mechanism is bearing the rectangular PEH itself (3). The latter is a commercially available device constituted by a 0.15 mm thick stainless steel substrate with two 0.254 mm thick PZT-5A piezoelectric layers deposited on its surfaces, having an overall 23 (length) × 15 (width) mm^2^ area [40]. The clamping mechanism allows the plectrum overlap over the PEH to be adjusted, while the clamping force is kept consistent by carefully tightening the two clamping bolts with a 1.5 Nm (±6%) torque by using a micro-torque wrench. The rotating speed of the motor, and thus, the plucking frequency, is controlled via a laboratory power supply (4) [41], while the output voltage generated by the PEH is measured via an Agilent^®^ DSO-X 2012A oscilloscope (5) [42] connected to the PEH electrodes via a TE^®^ 1051 resistance decade box (6) [43], set to the optimal load resistance of the PEH, thus enabling the assessment of the PEH power output [5,6,14].

### 3.2. Additive Manufacturing of the Plectra

Due to its versatility and rapid prototyping applicability [29,30], AM is employed in the herein considered original framework to produce the plectra with varying geometrical parameters. As the FDM AM technology enables the use of a wide array of materials [44], as well as to produce parts with a relatively high strength compared to other polymer-based AM technologies [30], it is chosen to produce plectra of different mechanical properties.

**Figure 4:**
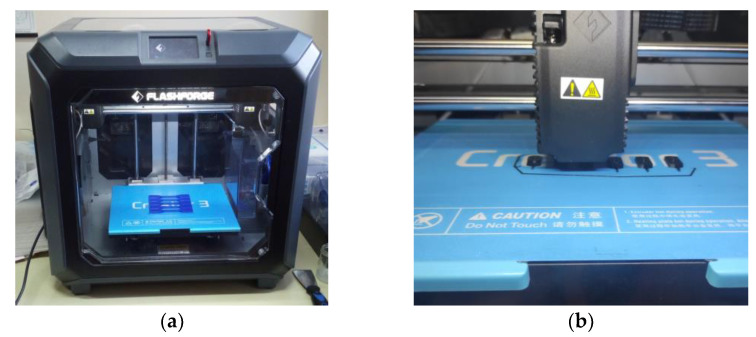
Additive manufacturing of the plectra: Flashforge^®^ Creator 3 3D printer [15] (**a**) and detail showing the rotor manufacturing process (**b**).

The FDM 3D printer used in this work is the Flashforge^®^ Creator 3 [44], shown in Figure 4, intended for both professional and industrial use, which comprises two *ϕ* = 400 μm independent extruders allowing to utilize a large number of filament materials, from the widely adopted acrylonitrile butadiene styrene (ABS) or polyethylene terephthalate (PET), to fiber-reinforced polymers. The machine uses a removable heated (up to 120 °C) base plate and it is able to produce 3D-printed parts within a 300 × 250 × 200 mm^3^ envelope, while allowing a positioning accuracy in the X-Y plane of 11 μm and of 2.5 μm along the Z axis [44]. The 3D-printed rotors bearing the plectra are manufactured so that all the individual parts are uniformly aligned during the printing process, ensuring their consistent mechanical properties. A substantial number of plectra with varying geometries and materials is, thus, manufactured quickly and cost effectively [34].

## 4. Initial Studies

The initial analyses of the effects of plectrum design parameters on PEH performances are conducted by using 3D-printed plectra of varying geometrical properties manufactured from different materials, namely ABS, polyamide (PA), polycarbonate (PC) and polylactic acid (PLA). As the stiffness of the plectrum, defined by its geometry and material properties, as well as the plucking speed have been identified in literature [26,27,28] as the most relevant factors in the plucking process, the values of the considered design parameters, i.e., of the second moment of area of the cross-section *I*_x_ and of the plectrum length *l*, as well as of the rotating speed *n* of the DC motor, are selected here within the ranges listed in Table 1. As stated above, these parameters are observed as continuous variables, while the type of material has a discrete character [34].

The required number of experiments is substantially reduced by using the LCVT DoE methodology [37,45], so as to create 20 random combinations of the values of the design parameter. The plectra with the hence defined geometries are then manufactured, and the experiments are carried out by employing the setup of Figure 3, allowing the measurement of the resulting voltage outputs. The powers generated by plucking the PEH with each of the used plectra is then calculated from the experimentally attained voltages and the set optimal resistances [5,6,14] by using the response surface methodology [34]. A quadratic regression model is, thus, generated for each of the considered materials with the reached coefficient of determination of *R*^2^ = 99.4%, describing, therefore, over 99% of the variance of the studied design parameters [34,46,47]. By employing the Generalized Reduced Gradient (GRG) code-based optimization algorithm [48], an optimal combination of the studied variable plectrum design parameters, resulting in the highest achievable power output values, can, therefore, be determined [34].

**Figure 5:**
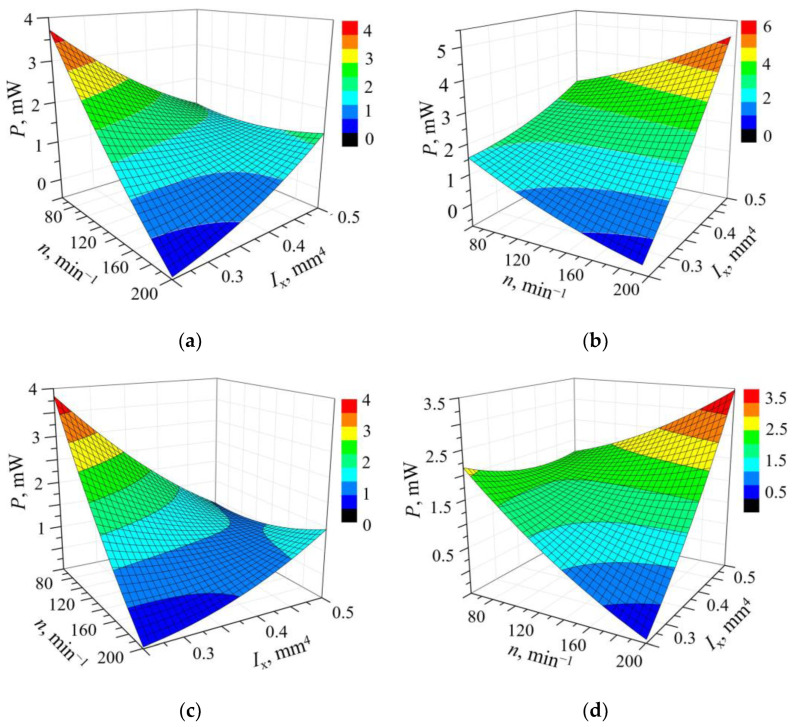
Power outputs resulting from the quadratic regression model for a constant plectrum length made from different materials: PA (**a**), PC (**b**), ABS (**c**) and PLA (**d**) [15].

The optimal plectrum design parameters and plucking speed, as well as the resulting maximal power output for each of the studied plectrum materials, are listed in Table 2. Since it is established that the optimal plectrum length is identical in all the studied cases and equal to the minimal length *l* = 9 mm, *l* is regarded as a constant, thus enabling a 3D graphical representation of the obtained results in the four response surface graphs, as shown in Figure 5.

By analyzing the depicted results, it can be noted that, despite having relatively comparable mechanical properties [29], the rotors made from different materials clearly give rise to different power outputs when used to pluck the same PEH, with the highest power values achieved by using the PC plectra, a material with tendentially the highest modulus of elasticity. A certain level of congruence between the optimal values of the design parameters and the considered limits of their value ranges can also be observed. In fact, the highest power outputs correspond to different combinations of the upper and lower limits of the values of *I*_x_ and *n*, so that in the case of PA and ABS, the highest powers are attained for the lowest *I*_x_ and *n* values, whereas in the case of PC and PLA the highest power outputs are obtained for the highest *I*_x_ and *n* values. On the other hand, all graphs seem to result in a shape with a saddle form, which could be an indication of multiple maxima outside of the ranges of values of the design parameters considered in the initial studies [34].

## 5. Investigation with Extended Parameter Ranges, Results and Discussion

Based on the initial results of Section 4, an extended study focused on plectra manufactured in the best performing plectrum material, i.e., polycarbonate (PC), and extending the range of design parameters values outside the previously studied one is carried out and presented in this section. Given the acquired experiences, the range of values of the plectrum length *l* is herein shifted. The new value ranges are, thus, those listed in Table 3. What is more, to avoid inconsistencies in the mechanical properties due to issues typical for AM technologies [49] and improve the repeatability of the experimental measurements, the number of plectra per rotor is reduced to one.

**Figure 6:**
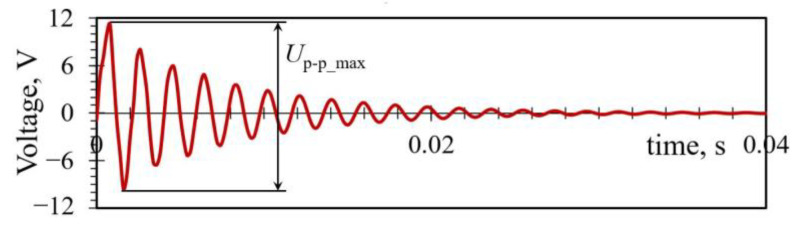
Peak-to-peak voltage used to calculate the maximal PEH power output *P*_p-p_max_.

The LCVT DoE algorithms are then employed again to generate 15 random combinations of the three considered design parameters, resulting in 15 single-plectrum rotor types with different geometries plucking the PEH at different rotor speeds. Five specimens of each rotor type are manufactured by using once more the Flashforge^®^ Creator 3 3D printer [44] and tested via the experimental setup described in Section 3.1. The thus attained maximal peak-to-peak voltage values *U*_p-p_max_ shown in Figure 6 are used to calculate, via the optimal load resistances the PEH is connected to [5,6,14], the respective power outputs *P*_p-p_max_, as reported in Table 4.

The standard deviation of the experimental results, listed in Table 4 as well, is found to be below ±0.6 mW, with the majority of the cases not exceeding ±0.2 mW, indicating a quite good measurement repeatability. By examining the results, it can also be observed that the highest measured power outputs are generated by the shortest plectra (*l* = 5 mm) and by an elevated plucking velocity (*n* = 311 min^−1^), possibly indicating a somewhat more pronounced impact of these parameters on the PEH power outputs.

As the initial study results clearly display a nonlinear behavior with an indication of a saddle-type shape in the graphs shown in Figure 5, a quadratic regression model—the simplest one suitable to describe a nonlinear behavior—is selected here. Such a model is fitted to the obtained experimental data by employing the response surface methodology [34], describing, therefore, via Equation (1), the effects of the studied design parameters on the maximum power output *P*_p-p_max_ of the plucked PEH. The predictive performance of this model is characterized by a coefficient of determination *R*^2^ = 90.43%, enabling the prediction of over 90% of the variance of the analyzed plectrum design parameters. Such an excellent reliability fully justifies, therefore, the adoption of the quadratic model.

*P*_p-p_max_ = 17.73 − 4.1∙*l*−4.5∙*I*_x_ + 0.0301∙*n* + 0.2831∙*l*^2^ + 9.22∙*I*_x_^2^ − 0.000041∙*n*^2^ − 0.889∙*l*∙*I*_x_ − 0.00192∙*l*∙*n* + 0.01223∙*I*_x_∙*n* (1)

**Figure 7:**
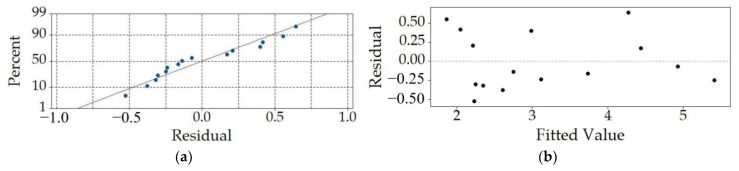

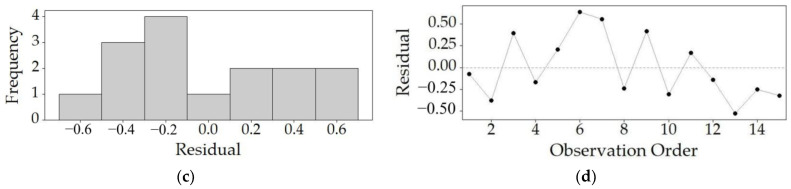
Residual analyses for *P*_p-p_max_: normal probability plot (**a**), residuals vs. the fitted value plot (**b**), frequency vs. residuals graph (**c**) and dependence of the residuals on the observation order (**d**).

As displayed in Figure 7, the developed regression model is then scrutinized with residual analyses to assess its predictive performances. It can, thus, be seen that the normal probability plot (Figure 7a) displays a good alignment with the normal probability line. The residuals vs. fitted value plot depicted in Figure 7b gives a good indication of the randomness of the residuals around the 0 line, indicating that the predictions have no or low bias. The quasi-normal distribution of the residuals is also evident in the histogram in Figure 7c. The variability of the residuals plotted against the order of the observations shown in Figure 7d, gives, in turn, an insight into the effect of the order of the experimental runs (observations), which does not seem to have an effect on the predictions of the model, as evident by the attained randomness of the depicted points. Thus, the performed analyses prove once more the validity of the used quadratic model.

**Figure 8:**
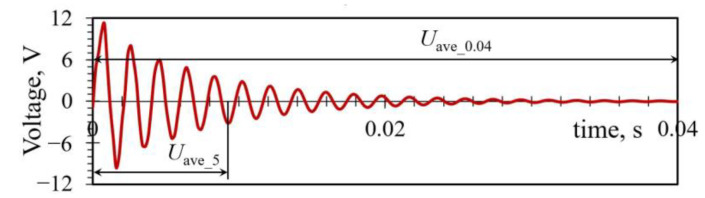
Average voltage values used to calculate different average PEH power outputs.

The experimental results are also studied in terms of the average power *P*_ave_5_, generated in the first five oscillations of the PEH, where the most prominent deflections, and thus, the highest voltage values *U*_ave_5_, occur, as well as in terms of the overall average output power *P*_ave_0.04_, generated by the harvester in the complete oscillation period *t* = 0.04 s (Figure 8).

A quadratic regression model is fitted to each of these average powers, providing detailed insights into the effects of plectrum design on the plucked responses of the PEH. The respective models of Equations (2) and (3) are characterized by, respectively, a coefficient of determination of *R*^2^ = 91.38% and *R*^2^ = 91.89%, both being, therefore, able to describe again over 90% of the variance of the analyzed parameters:

*P*_5p-p_ = 12.5 − 3.141∙*l* − 0.82∙*I*_x_ + 0.0231∙*n* + 0.2106∙*l*^2^ + 5.68∙*I*_x_^2^ − 0.000034∙*n*^2^ − 0.671∙*l*∙*I*_x_ − 0.00108∙*l*∙*n* + 0.00441∙*I*_x_∙*n* (2)

*P*_ave_ *=* 4.27 *−* 1.146*∙l −* 0.84*∙I*_x_
*+* 0.00859*∙n +* 0.0807*∙l^2^ +* 1.73*∙I*_x_*^2^ −* 0.000013*∙n^2^ −* 0.365*∙l∙I*_x_
*−* 0.000391*∙l∙n +* 0.00185*∙I*_x_*∙n* (3)

**Figure 9:**
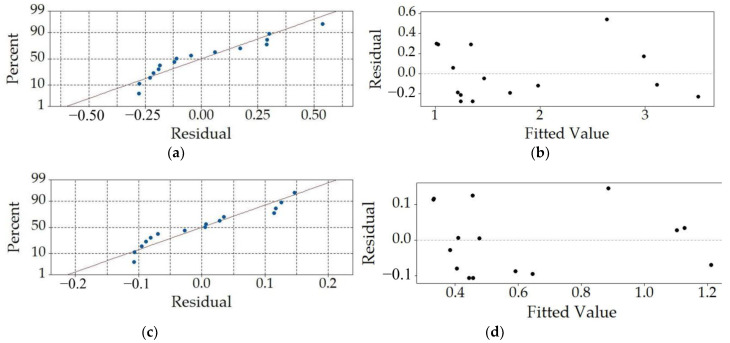
Residual analyses for *P*_5p-p_ and *P*_ave_0.04s_: normal probability plots (**a**,**c**) and residuals vs. the fitted value plots (**b**,**d**).

The respective most relevant residual plots are displayed in Figure 9a,b for the five peaks average model and in Figure 9c,d for the overall oscillation period average model. It can be observed here that in both cases there is a small number of points laying outside of the normal probability line, indicating the expected outliers that result in visible compromised randomness as the clustered residuals are close to the lower ends of the fitted values.

With the goal of finding the optimal combination of design and plucking parameters, resulting in the maximal power outputs, the models of Equations (1)–(3) are then subjected to an optimization process. Two different optimization algorithms are employed for this purpose, i.e., the Generalized Reduced Gradient (GRG) and the evolutionary algorithm.

The GRG method is based on examining the slope of the objective function while changing the input values; the optimal solution is achieved when the respective partial derivatives reach zero. While being a relatively fast optimization method, the resulting solution may not coincide with the global optimum, as the algorithm is likely to stop at a localized optimal value nearest to the initial conditions [48]. In the herein considered case, the parameters for the GRG algorithm are used with a multistart feature, where a population size of 100 randomly distributed initial values, with a chosen random seed of 0, is applied in starting the optimization process multiple times from different initial conditions, allowing a somewhat greater chance that the found solution is the global optimum.

The evolutionary algorithm, based on the theory of natural selection, is, on the other hand, a notably slower, but more robust optimization method, more likely to converge to the global optimal solution. The optimization process begins in this case with a random population of input value sets, and the results are evaluated in relation to the target value, with the set resulting in the solution closest to the latter selected to generate an offspring, which is a mutation of the best first-generation set. The process continues until the change between two consecutive generations is very small. As each member of the population in a generation is individually evaluated, the overall process is quite time consuming [48]. In the present case, the evolutionary algorithm is implemented with a convergence value of 5%—the maximum difference in objective values for the top 99% of the population of 100 value sets, with a random seed integer of 0. The maximum time without meaningful improvements in the objective value of the best solution in the population is, in turn, set to 30 s.

The resulting optimal values of the variable parameters, as well as their corresponding maximal power outputs, are then listed for the three considered cases in Table 5. It can be noted that the maximal achievable powers are significantly higher in the case of the optimal values of the design variables obtained by using the evolutionary algorithm, which is in accordance with the aforementioned drawback of the GRG method. In terms of the optimal design parameters, both methods clearly favor, however, in all the three cases the plectrum length coinciding with the lower limit of the selected range, i.e., *l* = 5 mm, confirming the tendency already seen in the initial studies of Section 4. When the second moment of area of the cross-section *I*_x_ is observed, the two optimization methods provide results on the opposite ends of the considered value range. The optimal plucking speed *n* provided by the GRG algorithm is notably lower than that resulting from the evolutionary algorithm, conforming again the issues described above. What is more, and particularly evident when the evolutionary algorithm is used, the value of *n* resulting in the highest average power outputs is considerably lower than the one resulting in the maximal peak-to-peak power. In this case, *n* resulting in the highest *P*_p-p_max_ value then coincides with the upper limit of its considered value range, i.e., *n* = 360 min^−1^.

**Figure 10:**
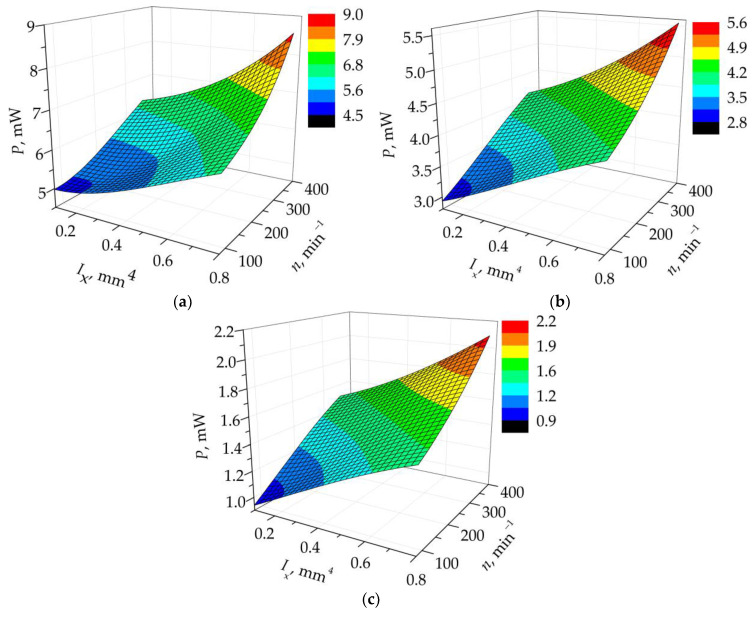
3D plots representing the effects of the studied design parameters on the responses of the plucked PEH for *l* = 5 mm: *P*_p-p_max_ (**a**), *P*_ave_5_ (**b**) and *P*_ave_0.04_ (**c**).

As the optimal plectrum length is identical in all the above cases, it can be considered as a constant, facilitating once more the graphical representation of the obtained results using 3D plots. The resulting graphs, displaying the effects of the variable design parameters *I*_x_ and *n* on the power outputs of an FUC-excited PEH derived by using the evolutionary optimization algorithm, are shown in Figure 10. It is clear here that a stiff plectrum design with a high value of the second moment of area of the cross-section and a short length, rotating at a high speed, will generate the highest possible outputs in terms of both the maximal as well as of the average powers. It is also clear that, contrary to the apparent indication during the initial studies, none of the graphs relating to the herein considered much broader range of values of the design parameters displays a saddle effect, but they all depict clear global maxima. Analogous to the initial studies, however, the highest output power values are attained when the design parameters are at the limits of their respective value ranges, indicating that an even stiffer plectrum design might induce higher power outputs; this could, in turn, induce obvious issues related to the (especially fatigue) strength of the PEH device itself.

## 6. Conclusions and Outlook

A first systematic investigation of the effects of the design of 3D-printed plectra on the responses of a plucked PEH is performed in this work. The basics principles of EH are provided, along with the distinctive issues of bimorph PEH devices used in environments with random excitations. The principles of the FUC-based excitation are outlined, as are the issues of the FDM AM technology and their effects on the mechanical properties of the thus manufactured parts.

To study systematically the effects of plectrum design and plucking parameters on PEH responses, random combinations of variables are then generated by using the DoE methodology, resulting in 15 different 3D-printed plectrum design configurations. These are tested by employing a specifically devised experimental setup, which allows assessing the PEH’s power outputs, studied in terms of the maximal peak-to-peak values, as well as of the average powers generated over the first five peaks and across the whole oscillation period. The obtained results are coupled with the respective design parameters, and a quadratic regression model is generated for each of the considered cases, always resulting in excellent predictive performances (*R*^2^ > 90%). An optimization process using two different algorithms is then employed with the objective of maximizing the PEH’s power output, allowing to establish a set of optimal design and plucking parameters.

The obtained results provide new insights into the plucking mechanism, showing that in all the cases, the models favor a short and fairly stiff plectrum, plucking the PEH at a relatively high speed, with the global optimal values of the design parameters clearly tending to coincide with the limits of the considered variable value ranges. If these limits were to be widened further, special attention would, however, have to be given to the strength of the piezoelectric layers to avoid their damage.

The optimal plectrum design and plucking conditions resulting from this research will be incorporated into an innovative FUC system that is a component of a wearable device, and tested and evaluated in laboratory as well as in factual working conditions. An improved plucking mechanism, which could facilitate the study of a larger number of parameters, will also be devised.

## Figures and Tables

**Table 1:** Considered ranges of values of the studied design parameters [34].

Parameter	Range of Values
*I*_x_, mm^4^	0.25–0.5
*l*, mm	9–13
*n*, min^−1^	60–200

**Table 2:** Maximal power outputs and optimal parameters for different plectrum materials [34].

	PA	PC	ABS	PLA
***P*** **_max_** **, mW**	3.72	5.06	3.85	3.46
***I*** **_x_** **, mm^4^**	0.25	0.5	0.25	0.5
***l*** **, mm**	9	9	9	9
***n*** **_0_** **, min^−1^**	60	200	60	200

**Table 3:** Extended value ranges of the studied parameters.

Parameter	Range of Values
*I*_x_, mm^4^	0.125–0.8
*l*, mm	5–10
*n*, min^−1^	95–360

**Table 4:** DoE design parameters values and resulting experimentally obtained maximal power values *P*_p-p_max_ with their standard deviations *σ*.

Design Parameters (DoE)			Results	
*l*, mm	*I*_x_, mm^4^	*n*, min^−1^	*P*_p-p_max_, mW	*σ*, mW
6	0.608	311	4.858	±0.362
7.5	0.125	360	2.229	±0.588
7.5	0.125	98	3.383	±0.198
7.5	0.792	360	3.571	±0.500
8	0.438	277	2.418	±0.176
6	0.258	302	4.917	±0.468
8	0.677	139	2.419	±0.121
7	0.226	172	2.877	±0.242
10	0.582	315	2.465	±0.117
9	0.688	214	1.941	±0.113
6	0.685	177	4.605	±0.116
8	0.692	308	2.608	±0.253
7	0.447	135	1.701	±0.048
5	0.240	159	5.161	±0.319
10	0.522	163	2.026	±0.179

**Table 5:** Design parameters resulting in the highest powers, obtained via the two considered optimization algorithms.

	GRG			Evolutionary		
	*P*_p-p_max_	*P*_ave_5_	*P*_ave_0.04_	*P*_p-p_max_	*P*_ave_5_	*P*_ave_0.04_
*l*, mm	5	5	5	5	5	5
*I*_x_, mm^4^	0.125	0.125	0.125	0.8	0.8	0.8
*n*, min^−1^	268.64	268.40	264.09	360	312.03	312.12
*P*, mW	6.29	4.08	1.37	8.64	5.67	2.14

## Data Availability

The experimental data presented in this work are available online at https://repository.riteh.uniri.hr/islandora/object/riteh:3563 (accessed on 20 February 2023).
